# Fluorescence Guided Surgery in Gastric Cancer: What Do We Have and What Can We Do?

**DOI:** 10.1002/ags3.70053

**Published:** 2025-06-13

**Authors:** Chun Zhuang, Han‐Kwang Yang

**Affiliations:** ^1^ Department of Gastrointestinal Surgery, Renji Hospital Shanghai Jiao Tong University School of Medicine Shanghai China; ^2^ Division of Gastrointestinal Surgery, Department of Surgery Seoul National University Hospital Seoul South Korea; ^3^ National Cancer Center Goyang‐si Korea

**Keywords:** blue dye, carbon particle, gastric cancer, gastroesophageal junction cancer, indocyanine green

## Abstract

**Background and Objective:**

Fluorescence imaging has emerged as a valuable adjunct in gastric surgery, enhancing resection precision and oncologic outcomes. However, the use of indocyanine green (ICG) remains controversial due to uncertainties in efficacy and administration. A lack of standardized protocols persists. This review summarizes current applications of fluorescence in gastric cancer surgery, outlining existing challenges and future research needs.

**Methods:**

A systematic PubMed search (2004–2024) was conducted using keywords such as “indocyanine green,” “carbon particle,” “blue dye,” “gastric cancer,” and “gastroesophageal junction cancer” to identify and review key uses of fluorescence agents in gastrointestinal malignancies.

**Key Findings:**

Fluorescence‐guided imaging aids intraoperative tumor localization, shortens operative time, and enhances lymph node (LN) yield, improving staging accuracy. Its role in sentinel lymph node (SLN) detection is still under debate due to false negatives. ICG fluorescence angiography (ICG‐FA) may lower anastomotic leak rates, though strong supporting evidence is limited. No consensus exists regarding ICG dosage, timing, or delivery method.

**Conclusions:**

Current evidence supports the safety and efficacy of fluorescence imaging in gastrointestinal surgery, with promising outcomes in precision and staging. However, uniform protocols for fluorescence use are urgently needed. Future studies should aim to standardize administration and optimize clinical implementation to fully realize its benefits.

## Abstract 2

This review explores the current applications of fluorescence‐guided surgery in gastric cancer, focusing on tracers such as indocyanine green (ICG), blue dyes, and carbon particles. It highlights their roles in tumor localization, lymph node mapping, and perfusion assessment, while emphasizing the need for standardized protocols to optimize surgical precision and outcomes.

## 1 Introduction

According to recent global cancer data, gastric cancer is the fifth most common malignancy and the fourth leading cause of cancer‐related death [1]. Despite advances in endoscopic resection, chemotherapy, radiotherapy, immunotherapy, and targeted therapy, surgery remains the mainstay. Since Kitano first reported laparoscopic radical gastrectomy, its safety and efficacy have been confirmed over the past two decades [2, 3]. With ongoing technological innovations, laparoscopic surgery has gained prominence. To improve outcomes, surgical oncology principles including achieving negative margins and complete lymph node (LN) dissection must be upheld [4]. Yet, key challenges in minimally invasive gastric cancer surgery persist, including tumor localization, perfusion assessment, and adequate LN harvest. Fluorescent contrast agents now offer real‐time visualization to delineate tumors and metastatic sites more precisely [5]. This review explores current applications and future directions of fluorescent agents in gastric cancer surgery.

## 2 Tracers in Gastrectomy

### 2.1 Blue Dyes

Blue dyes such as methylene blue, isosulfan blue, and patent blue are commonly used in gastrectomy for sentinel lymph node (SLN) biopsies. Methylene blue, a small aromatic compound, is one of the earliest blue tracers, approved by the FDA for its visibility and use with formalin‐fixed LN harvesting [6]. Isosulfan blue, a triphenylmethane dye, was the first FDA‐approved agent for lymphangiography and is delivered via endoscopic or serosal injection [6]. Patent Blue (V/E131), also a triphenylmethane dye, is widely used in SLN mapping for gastric cancer surgery [7]. These dyes are valued for low cost, accessibility, and ease of use, with sulfonic acid groups promoting lymphatic targeting. However, they have drawbacks such as poor quantifiability, rapid diffusion, degradation, and allergy risk [8].

### 2.2 Carbon Particles

In gastrectomy, carbon particle use has advanced from traditional Indian inks to carbon nanoparticle suspension injection (CNSI), favored for its simplicity, low cost, and safety. With a 150 nm diameter, CNSI matches the 100–500 nm size of lymphatic capillary gaps, enabling selective lymphatic uptake over blood vessels. This ensures efficient lymph node (LN) mapping and identification [9]. CNSI offers prolonged LN retention, affordability, and accessibility. However, it may blacken the surgical field, complicating visualization.

### 2.3 Indocyanine Green (ICG)

ICG binds rapidly to plasma proteins and bilirubin, promoting swift lymphatic uptake. Beyond visual use, it enables infrared and fluorescence imaging, making it a key contrast agent. ICG fluoresces under NIR light (750–810 nm), peaking at 830 nm after stimulation, and is detected by advanced imaging systems [10]. Devices like NIFI, IREE, and IRLS enhance tissue visualization. Systems such as Hyper Eye, D‐light P, and PINPOINT efficiently detect ICG fluorescence under white light [11]. Infrared‐based methods, especially IREE and IRLS, outperform traditional systems in sensitivity, with IREE showing the highest recognition rates [12].

### 2.4 Radioactive Tracers

Various radioactive tracers are used for LN mapping, notably technetium‐99m (99mTc) compounds like antimony sulfur, tin, and sulfur colloids. Indium‐111 pentetreotide and gallium‐68‐DOTA‐peptides are also applied in gastroenteropancreatic neuroendocrine tumor surgeries [13, 14]. These tracers localize in SLNs within 2 h and persist over 20 h due to macrophage uptake. 99mTc is typically injected submucosally 1 day preoperatively (2 mL into four quadrants) [15]. Benefits include low allergy risk, easy digitization, and long SLN retention. Limitations include inability to trace lymphatics, high cost, radiation exposure, lack of visual feedback, background scatter, legal and supply issues, and equipment demands [16]. Thus, they are rarely used alone in surgery.

### 2.5 Dual Tracers

To overcome radiotracer limitations in lymphatic mapping and false positives from low‐molecular‐weight dyes, dual tracers are often used. Radioactive tracers are injected a day before surgery, followed by intraoperative submucosal blue dye or ICG, with SLNs identified via visual and gamma detection [17]. ICG's efficacy depends on NIR or fluorescence imaging, explaining its reduced performance when used alone. Only one trial has combined ICG's NIFI capabilities with radioisotopes [18]. Dual tracers improve LN detection as they are complementary; one study showed nodes identified as hot or dyed were 2.5 times more than those identified by both criteria [19]. Kong et al. evaluated novel NIR tracers, NIR‐PNG and ICG/c‐PGA, which reduced dispersion and prolonged nodal retention compared to traditional ICG [20]. Though tested in large animals, these tracers show promise for sentinel node surgery in gastric cancer, warranting human trials to confirm safety and efficacy.

### 2.6 Cancer Specific Targeted Tracer

Monoclonal antibody‐based fluorescent agents have advanced intraoperative imaging by enabling real‐time tumor margin delineation and small lesion detection. They also support targeted therapy through antibody‐drug conjugates. Current research targets antigens such as CEA, CA19‐9, EGFR, HER2, and prostate‐specific membrane antigen. SGM‐101, combining BM104 dye with anti‐CEA antibody, showed promise for fluorescence‐guided surgery (FGS), though low CEA expression in gastric cancer limits its utility [21]. Terwisscha van Scheltinga et al. conjugated trastuzumab with IRDye 800CW, achieving sensitive in vivo tumor detection [22]. Jeong et al. reported peak liver/spleen signals at 24 h and tumor signals at 72 h post‐injection, confirming high HER2 affinity [23]. Affibody‐IRDye800CW also bound HER2 but lacked tumor signals due to rapid clearance. Cheng et al. used an RGD‐ICG probe in gastric cancer peritoneal metastasis, achieving 93.93% sensitivity and specificity, 1.8 mm lesion detection, and a 3.26‐fold reduction in operative time [24].

### 2.7 Nerve Specific Targeted Tracer

With improved endoscopic screening and treatment, gastric cancer prognosis has advanced in East Asia. Surgeons now aim to enhance quality of life while ensuring oncologic safety. Laparoscopic pylorus‐preserving gastrectomy (LPPG) has become popular. Wang et al. showed that preserving the hepatic branch of the vagus nerve during LDG and LPPG reduces postoperative gallstones [25]. Nerves are usually identified by anatomy or electromyography, but small or buried ones may be missed. Whitney et al. used phage display to find nerve‐binding peptides, visualizing nerves in mice within 2 h with 8‐h contrast and no toxicity [26]. Gonzales demonstrated that Hsp1a, targeting Nav1.7 channels, could label peripheral nerves intraoperatively [27]. Wu identified NP41, enabling rapid visualization of live and degenerate nerves in mice [28]. Hingorani introduced HNP401, a fluorophore‐labeled peptide highlighting various human nerves, showing potential for surgical application [29].

## 3 Application of Fluorescence Imaging Technology in Gastrectomy

### 3.1 Navigation for Metastatic LN

#### 3.1.1 Assessment of SLN

For cT1 cases, D2 or D1 lymph node dissection may be unnecessary due to excessive trauma and impaired gastric function. To preserve function while ensuring oncologic outcomes, reducing surgical trauma is essential. The stomach's sentinel lymph node (SLN), as the primary drainage point, is most likely to harbor metastasis. SLN navigation remains controversial due to micrometastasis and skip patterns. Accurate SLN identification is a key focus. Methylene blue injected into submucosa, subserosa, or gastric arteries reaches SLNs within 5–10 min [8, 30, 31]. Isosulfan blue achieves 90%–100% detection, with high sensitivity (~97%), and an average of 3 SLNs and 25 total LNs retrieved, though false negatives remain due to complex drainage or vessel disruption in larger tumors [32].

ICG fluorescence imaging (ICG‐FI) offers high SLN detection accuracy for T1 and 95.0% for T2 gastric cancer [33]. Many studies confirm > 90% detection with excellent sensitivity in early‐stage disease [34]. For negative SLNs, function‐preserving surgery (FPS) with lymphatic basin dissection (e.g., wedge, segmental, or limited gastrectomy) is advised [35]. Compared to standard D2 or D1 gastrectomy, basin dissection (D0) minimizes tissue damage while ensuring efficacy. ICG‐FI enables precise staging and planning. However, concerns about oncologic safety and biopsy standardization persist. The Korean SENORITA trial found similar complication rates between sentinel node surgery and standard gastrectomy [36]. A phase II trial showed 96% relapse‐free and 98% overall 3‐year survival for SLN‐negative patients undergoing limited resection. A cohort study reported 5‐year survival of 96.8% and recurrence of 0.43% with navigation surgery, versus 91.3% and 1.30% in controls [37]. While outcomes are promising, more data are needed to confirm safety.

#### 3.1.2 LNs Mapping for AGC

In resectable advanced GC, radical resection and sufficient LN retrieval are essential. ICG‐FI has been shown to improve LN harvesting during gastrectomy, with multiple studies reporting increased LN yields compared to traditional techniques [38, 39]. No major differences in perioperative or long‐term outcomes have been noted between ICG and non‐ICG groups [38].

ICG‐FI enables clear mapping of lymphatic drainage, improving LN retrieval and reducing blood loss, especially in the challenging infrapyloric region. Park et al. showed that NIR guidance enhances safety, reduces bleeding, and benefits less experienced surgeons during infrapyloric dissection [40]. ICG‐FI also effectively detects splenic hilar nodes with high negative predictive value, helping determine whether dissection is necessary [41]. This technique clarifies complex lymphatic patterns and aids in identifying skip metastasis, thus optimizing dissection strategies and outcomes.

However, ICG‐FI has limitations. In advanced stages (T3–T4), false‐negative rates rise. Contributing factors include suboptimal injection, tumor‐induced lymphatic blockage, and variability in histologic assessment. Additionally, gastric lymphatic complexity hinders full metastatic node detection. While ICG‐FI enhances visualization, its limited specificity and risk of false negatives remain barriers to fully optimized lymphadenectomy.

### 3.2 Tumor Localization and Resection Margin

In curative gastric surgery, recommended resection margins are ≥ 3 cm for expansive, ≥ 5 cm for infiltrative T2+, and ≥ 2 cm for T1 tumors. The resection extent and reconstruction method impact early morbidity and long‐term quality of life [42]. Minimally invasive surgery improves recovery and QoL, but tumor localization remains difficult unless visible on the serosa. Surgeons rely on monitor images or forceps feedback, which can be imprecise. Fluorescent tracers offer superior accuracy, safety, and usability in surgical oncology [43].

Intraoperative gastroscopy with methylene blue or indigo carmine enables accurate tumor marking. Xuan et al. used this method in laparoscopic gastrectomy, achieving complete negative margins [44]. Patent blue with sodium hyaluronate has also ensured clear margins, guided by endoscopic clips [7]. NIR fluorescence has extended methylene blue's use in tumor localization. Although it targets gastric tissues more than tumors, it aids in defining resection margins [45].

Indian ink submucosal injection helps serosal tumor identification, though 21% of proximal margins were shorter than expected, especially in aggressive tumors. ICG injection, followed by NIR or robotic visualization, enables accurate margin delineation and reduces operative time [43]. ICG‐FI can secure ≥ 28 mm margins, aiding in both oncologic safety and functional preservation [46].

Endoscopic fluorescent clips also assist localization but have limited penetration and weaker signal, requiring camera or tissue adjustments [47]. Fluorescence shortens tumor identification time. In a study of 93 gastric cancer patients, the ICG group showed a significantly shorter operative time (235 vs. 275 min, *p* = 0.006) with no margin difference, supporting its role in improving surgical efficiency and reducing complications [48].

### 3.3 Evaluation of Bowel Perfusion

Anastomotic leakage (AL) is a serious complication in gastric cancer surgery, with reported rates of 1.2%–6.7%, contributing to increased mortality and poor outcomes [49, 50, 51]. Unlike colorectal surgery, upper GI leaks lack diversion options, often resulting in severe nutritional deficits. Key factors for successful anastomosis include adequate perfusion, minimal tension, precise tissue alignment, and reduced contamination risk [52]. Traditionally, perfusion is evaluated visually or with tools like Doppler and fluorescein, though both have limitations in consistency and accuracy.

ICG fluorescence angiography (FA) offers real‐time perfusion assessment. Huh et al. found that, despite favorable clinical indicators, AL still occurred when localized hypoperfusion was evident in NIR review, suggesting ICG FA's predictive value [53]. Mori et al. later identified that delayed ICG appearance on one side of the anastomosis predicted leakage [54].

Despite its promise, ICG FA interpretation is subjective. Blue dye provides immediate visual confirmation, reducing inter‐observer variability. Its integration with ICG FA and hyperspectral imaging (HSI) may enhance perfusion evaluation. In laparoscopic RYGB, blue dye detected the risk of leak even when ICG perfusion seemed sufficient, guiding the reinforcement of the anastomosis [55]. While lacking quantitative precision, blue dye's simplicity and reliability make it a valuable adjunct in intraoperative decision‐making, improving anastomotic integrity and recovery.

### 3.4 Facilitate Surgical Dissection

In laparoscopic surgery for remnant gastric cancer (RGC) post‐distal gastrectomy with Billroth‐I reconstruction, dense adhesions and distorted anatomy complicate organ boundary identification. Under white light, dissection is difficult and risky. Real‐time ICG fluorescence imaging (ICG‐FI) improves visualization, enabling safer dissection. Yamazaki et al. reported two LTG cases for RGC without complications using ICG‐FI, which clearly delineated the liver and bile duct, aiding safe layer separation [56].

An aberrant left hepatic artery (ALHA), arising from the left gastric artery (LGA), occurs in 6.5%–34% of patients [57, 58]. During radical gastrectomy, the LGA is usually ligated, which may compromise ALHA flow and lead to hepatic complications. Preserving the ALHA is ideal but not always feasible. Lee et al. introduced a simple NIR fluorescence imaging (NIRFI) technique to visualize ALHA territories intraoperatively, allowing informed preservation or ligation decisions without postoperative liver dysfunction [59].

## 4 Technical Aspect of Tracer Administration

Patent blue is commonly injected as 0.2 mL of a 2% solution into four quadrants around the tumor during intraoperative endoscopy; higher doses are used for subserosal or intramuscular injection [60, 61]. The dye appears on the serosal surface shortly after submucosal injection, and SLNs become visible within 16 min [60]. The staining lasts up to 2 h. Methylene blue is injected into the submucosa, subserosa, or gastric arteries, with SLN detection typically within 5–10 min [62]. CNSI, the preferred carbon tracer, is injected submucosally 6–48 h before surgery [63].

Cancer‐specific tracers, such as IRDye 800CW‐labeled bevacizumab and trastuzumab, show high specificity in preclinical models, peaking 2–3 days post‐injection [23]. A CEA‐targeted single‐chain antibody tracer achieved peak contrast at 72 h [21]. Nanobody tracers like EpCAM‐F800 offer rapid tumor labeling within 4–8 h and imaging duration up to 96 h [64].

**FIGURE 1:**
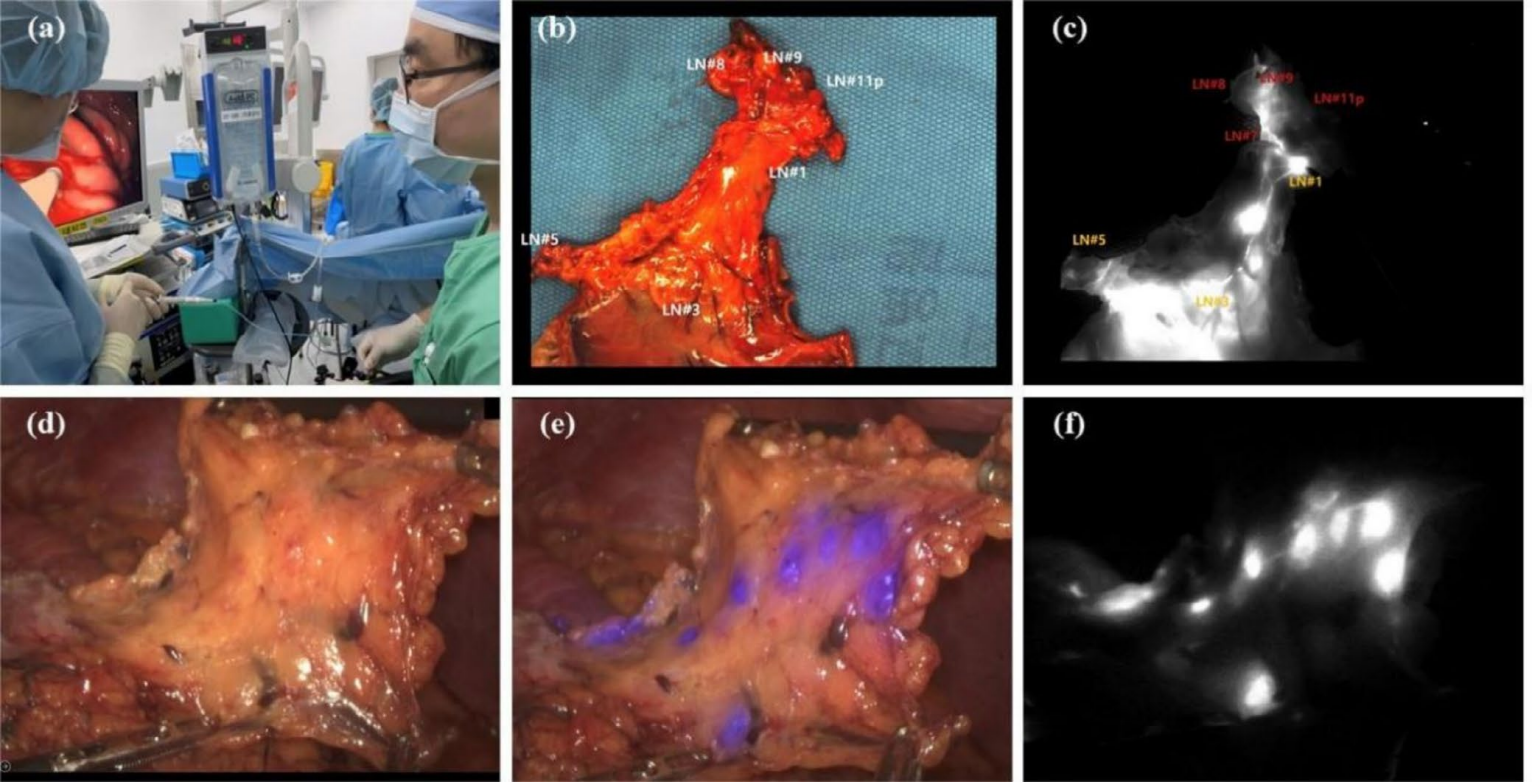
Intraoperative ICG injection via gastroscope is an effective method for surgeons to gain familiarity with endoscopic procedures (a), offering relatively accurate lymph node mapping (b‐c) and significantly enhancing the precision of real‐time lymphatic mapping compared to preoperative methods (d‐f).

ICG dose optimization studies suggest 0.625–1.25 mg/mL concentrations, with lower doses (0.5–0.05 mg/mL) providing higher sensitivity [65]. Advanced systems like da Vinci Xi and PINPOINT allow reduced concentrations. Typically, 0.5–0.6 mL is injected into four quadrants [66]. Subserosal injection offers cost and convenience advantages over submucosal methods [67]. Preoperative ICG provides stable, prolonged fluorescence and enhances lymph node retrieval but may be affected by scheduling delays. Intraoperative injection under laparoscopic guidance avoids peritoneal spillage and enables real‐time lymphatic mapping with improved safety and surgical precision. In contrast, intraoperative ICG injection via gastroscope offers the advantage of minimizing peritoneal spillage, as it is guided by laparoscopy. This technique also serves as an effective method for surgeons to gain familiarity with gastroscopic procedures. Moreover, when performed under intraoperative anesthesia and monitoring, ICG injection is safer, even in the rare event of anaphylaxis. Additionally, this approach allows for more precise real‐time adjustments and immediate lymphatic mapping, which can significantly enhance dynamic decision‐making during surgery (Figure 1).

## 5 Future Directions for Fluorescence‐Guided Gastrectomy

A major innovation in gastric cancer surgery is the development of fluorescent agents with improved specificity and safety. While ICG is widely used for SLN mapping and lymphatic tracing, its limitations include nonspecific tumor binding and false negatives. Future research aims to enhance detection accuracy and staging through more specific lymphatic tracers, improving surgical precision and outcomes.

**FIGURE 2:**
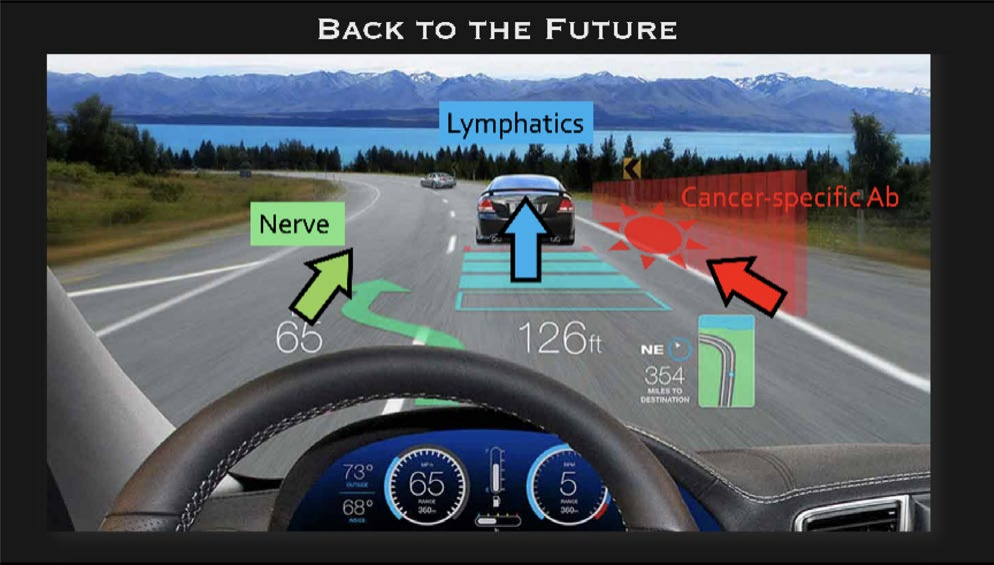
In gastrectomy, ICG maps drainage routes like GPS while cancer‐specific fluorescence detects tumor margins like onboard radar, synergistically guiding resection boundaries. Nerve‐specific tracers act as collision alerts, identifying safe zones for nerve preservation, optimizing safety and navigation precision.

Cancer‐specific antibody‐based fluorescent agents offer potential for precise tumor targeting and real‐time visualization, aiding in micro‐metastasis detection and minimizing surgical extent. This enhances resection completeness while preserving healthy tissue. Additionally, nerve‐specific tracers enable real‐time identification of critical nerves during surgery, supporting nerve‐sparing techniques and reducing postoperative complications like delayed gastric emptying. The integration of these advanced fluorescent agents into surgical practice has the potential to significantly enhance the safety, efficacy, and overall outcomes of gastric cancer surgeries (Figure 2).

## 6 Conclusion

Fluorescent agents like ICG and methylene blue (MB) enhance surgical precision by providing real‐time visualization of tumor margins and metastases, aiding complete resection and improved outcomes. Fluorescence imaging has shown value in lymph node navigation, particularly with ICG‐FI for sentinel lymph node detection, allowing tailored lymphadenectomy with reduced morbidity and preserved oncologic safety. However, challenges remain in standardizing SLN biopsy techniques and confirming the long‐term safety of limited surgery. Future efforts should focus on developing more specific tracers and integrating fluorescence with robotic‐assisted surgery to further improve surgical accuracy and patient outcomes.

## Author Contributions

**Chun Zhuang:** investigation, writing – original draft, methodology, writing – review and editing. **Han‐Kwang Yang:** conceptualization, writing – review and editing, methodology.

## Conflicts of Interest

The authors declare no conflicts of interest.
